# LINC01355 Contributes to Malignant Phenotype of Oral Squamous Cell Carcinoma and Cytotoxic T Cell Infiltration via Activating Notch Signaling Pathway

**DOI:** 10.1155/2021/1830790

**Published:** 2021-07-27

**Authors:** Chen Zou, Siyuan Wu, Haigang Wei, Hailing Luo, Zhe Tang, Xia Li, Xiaozhi Lv, Yilong Ai

**Affiliations:** ^1^Foshan Stomatological Hospital, School of Medicine, Foshan University, Foshan, Guangdong, China; ^2^Department of Oral & Maxillofacial Surgery, NanFang Hospital, Southern Medical University, Guangzhou, China

## Abstract

LINC01355 has been demonstrated to be dysregulated in several cancers. However, the exact molecular function of LINC01355 in the pathogenesis of OSCC remains unstudied. Here, we reported the effect of LINC01355 in OSCC and investigated the mechanisms. Firstly, we found that the results indicated LINC01355 was increased in OSCC cells. Knockdown of LINC01355 repressed OSCC cell proliferation, migration, and invasion. Recently, immunotherapy is a significant method for the treatment of cancers, in which CD8+ T cells exhibit a significant role. The influence of LINC01355 on the antitumor activity of CD8+ T cells was also focused in this study. As shown, the silence of LINC01355 could repress OSCC tumor growth via inducing CD8+ T cell immune responses. In addition, we found that downregulation of LINC01355 significantly restrained CD8+ T cell apoptosis, induced CD8+ T cell percentage, and enhanced the cytolysis activity when cocultured with OSCC cells. It has been reported that the Notch pathway represses CD8+ T cell activity in cancer patients. In our present study, we displayed that lack of LINC01355 suppressed OSCC malignant behaviors and enhanced the antitumor activity of CD8+ T cells via inactivating Notch signaling. We showed that decreased LINC01355 significantly restrained the Notch signal via a decrease of Notch-1, JAG-1, and HES-1. Repression of Notch1 reversed the effect of LINC01355 in OSCC cells. In conclusion, it was implied that LINC01355 might induce the development of OSCC via modulating the Notch signal pathway, which could provide a candidate therapeutic target for OSCC.

## 1. Introduction

Oral squamous cell carcinoma (OSCC) can account for almost 90% of malignancy in the oral cavity around the world [1, 2]. Great progresses have been made in treatments including surgery, chemotherapy, and radiotherapy during the last few years. However, the 5-year overall survival of OSCC patients is quite low [3–5]. Therefore, the underlying mechanism of OSCC is required to reduce the morbidity and mortality of OSCC.

LncRNAs are common transcripts with more than 200 nucleotides [6–8]. Evidence has reported lncRNAs can exert a crucial role in modulating genes at various levels in the biological processes of tumors [9, 10]. For instance, antisense lncRNAs hybridize to specific pre-mRNA and this can result in the alternatively spliced mRNAs or endogenous siRNAs [11]. In addition, lncRNAs serve as a sponge to inhibit microRNA expression [12]. Furthermore, lncRNAs can lead to dysregulated localization within cells through interacting with proteins [13].

As reported in recent years, the progression of OSCC is a complicated process. The role of lncRNAs has been demonstrated, which reveals lncRNAs can function a lot in OSCC. For example, HOXA11-AS can induce OSCC development through sponging miR-98-5p and inducing YBX2 [14]. FAL1 promotes OSCC development through modulating miR-761 and CRKL [15]. In addition, lncRNA SNHG5 contributes to OSCC cell progression through sponging miR-655-3p and FZD4 [16]. Nevertheless, there are few studies on the correlation between LINC01355 and OSCC.

In many cancers, Notch signaling is dysregulated and plays crucial roles in multiple cancers through modulating cancer cell processes. For instance, DCST1-AS1 sponges miR-92a-3p to enhance Notch1 in endometrial carcinoma [17]. LncRNA NEAT1 promotes OSCC progression via Notch signaling [18]. In addition, lncRNA HNF1A-AS1 induces the development of OSCC via the activation of the Notch pathway [19]. However, the detailed function of Notch in OSCC is poorly known.

Currently, the biological mechanism of LINC01355 in OSCC was studied. We reported that LINC01355 was highly upregulated in OSCC tissue and cells. We found that LINC01355 was involved in OSCC progression and cytotoxic T cell infiltration through activating the Notch pathway.

## 2. Methods and Materials

### 2.1. Cell Culture

HOMEC cells were purchased from Shanghai Bioleaf Biotechnology. CAL27, TSCCA, SCC-15, Tca8113, SCC-7, and SCC-9 cells were purchased from the ATCC (Manassas, VA, USA). The cells were maintained in DMEM with 10% FBS, 100 mg/mL penicillin G, and 100 U/mL streptomycin in an incubator (37°C, 5% CO_2_).

### 2.2. Isolation of CD8+ Effector T Cells

EasySep ™ Direct Human CD8+ T cell Isolation Kit (STEMCELL, Cambridge, MA, USA) was used to isolate CD8+ T cells from the whole blood of healthy individuals. Isolated cells were incubated with RPMI-1640 medium with 10% FBS.

### 2.3. Cell Transfection

To construct cell line with LINC01355 overexpression, LINC01355 cDNA was amplified by PCR and inserted into the pEGFPC3 vector to establish the LINC01355 overexpression vector. The suppression of LINC01355 and Notch1 was carried out by shRNA interference (GenePharma, Shanghai, China).

### 2.4. CCK-8 Assay

Transfected OSCC cells seeded in 96-well plates were incubated for indicated days. Then, the cells were subjected to CCK-8 (Dojindo, Tokyo, Japan) and incubated for 2 h. After 2 hours, the optical density was tested at 450 nm.

### 2.5. Colony Formation Assay

In detail, 400 cells were seeded into 6-well plates for two weeks. Afterwards, cells were washed by 1x PBS, fixed by 4% formaldehyde, stained using 0.1% crystal violet, and recorded using EOS 90D.

### 2.6. EdU Staining

BeyoClick™ EdU Cell Proliferation Kit was carried out to test cell proliferation. EdU solution was added and incubated for 2 h. 4% paraformaldehyde fixing solution was added for 20 min, and 0.5% Triton X-100 was used. Next, 100 *μ*L Apollo reaction solution was used and Hoechst33342 (Beyotime, Shanghai, China) was used for 30 min. Fluorescence microscopy (Nikon) was used to capture the images, and the images were merged by the Adobe Photoshop 6.0 software.

### 2.7. Transwell Assay

Transwell assay was used to test cell invasion and migration. 3 × 10^5^ OSCC cells were transferred to the upper Matrigel-coated or not Matrigel-coated invasion chambers in a serum-free DMEM medium. The lower chambers were loaded with DMEM medium with 10% FBS. After 24 h, we removed the nonmigrated or noninvaded cells on the upper surface. The cells on the underside surface were fixed by 4% paraformaldehyde, stained using 0.1% crystal violet, and imaged using a microscope.

### 2.8. Apoptotic Analysis

Cell apoptosis was analyzed by an Annexin V-FITC/PI apoptosis detection kit (Keygen, Nanjing, China). In brief, 1 × 10^6^ transfected OSCC cells were plated into 6 well plates. After trypsinization, 5 *μ*L Annexin V-FITC and then 5 *μ*L PI solution were added to the cells in dark. Flow cytometric analysis was conducted using a FACSCalibur flow cytometer.

### 2.9. LDH Cytotoxicity Assay

CyQUANT ™ LDH Cytotoxicity Assay Kit was used to assess the cytotoxicity of CD8+ T cells. Isolated effector CD8+ T cells were cocultured with target OSCC cells. The conditioned medium was transferred into a 96-well plate. After adding stop solution, OD490 and OD680 values were tested and the difference (OD490-OD680) indicated the LDH activity.

### 2.10. Proliferation of CD8+ T Cells

The proliferation of CD8+ T cells cocultured with OSCC cells was tested by CFSE labelling. Effector CD8+ T cells were stimulated using anti-CD3 and anti-CD28. Cells were labeled with CFSE and cocultured with target OSCC cells.

### 2.11. Real-Time PCR

Trizol was carried out to extract total RNA. Total RNA was reversely transcribed into cDNA using Takara reverse transcription system (Dalian, China). qPCR analysis was carried out with Applied Biosystems 7500 Real-Time PCR System (Thermo Fisher Scientific, Waltham, MA, USA) using iQ ™ SYBR ® Green Supermix Kit. Primers were purchased from Sangon Biotech and listed in Table 1.

### 2.12. Western Blot

RIPA buffer (Sangon Biotech) was used to extract total protein. After separated using SDS-PAGE, proteins were transferred onto PVDF membranes, then blocked 1 h. The membranes were incubated with primary antibodies (Cell Signaling Technology, Danvers, MA, USA): Notch1, JAG1, and HES-1 or GAPDH at 4°C for a whole night. After HRP-conjugated anti-rabbit secondary antibody was used to incubate the membranes for 2 h, the signal was evaluated using the ECL system.

### 2.13. Tumor Xenografts

12 female BALB/c mice aged 6 weeks were acquired from the Animal research center of the Chinese Academy of Sciences (Shanghai, China). Mice were grouped into two groups (*n* = 6): sh-NC group and sh-LINC01355 group. Mice were injected with SCC-7 cells infected with sh-NC or sh-LINC01355. Tumor weight was measured by a vernier caliper, and we recorded the volume every 3 days. 22 days later, we sacrificed the mice. The animal assays were approved by the Ethics Committee of Foshan Stomatological Hospital, School of Stomatology and Medicine, Foshan University.

### 2.14. Flow Cytometry Analysis

To assess the frequencies of IFN-*γ*+ and TNF-*α* in CD8+ T cells, tumor cells were fixed, permeabilized, and stained by anti-CD8-APC, anti-IFN*γ*-FITC, or anti-TNF-*α*-FITC (BD Biosciences, San Jose, California, USA). Then, staining with fluorophore-conjugated secondary antibodies was used for flow cytometry analysis.

### 2.15. Statistical Analysis

Data were analyzed using SPSS19.0, and all the data were presented by mean ± SD method. The difference was analyzed by Student's *t*-test (two groups) or two-way ANOVA (multiple groups). *p* value less than 0.05 was considered as significant threshold.

## 3. Results

### 3.1. Loss of LINC01355 Inhibited OSCC Cell Proliferation and Induced Cell Apoptosis

**Figure 1:**
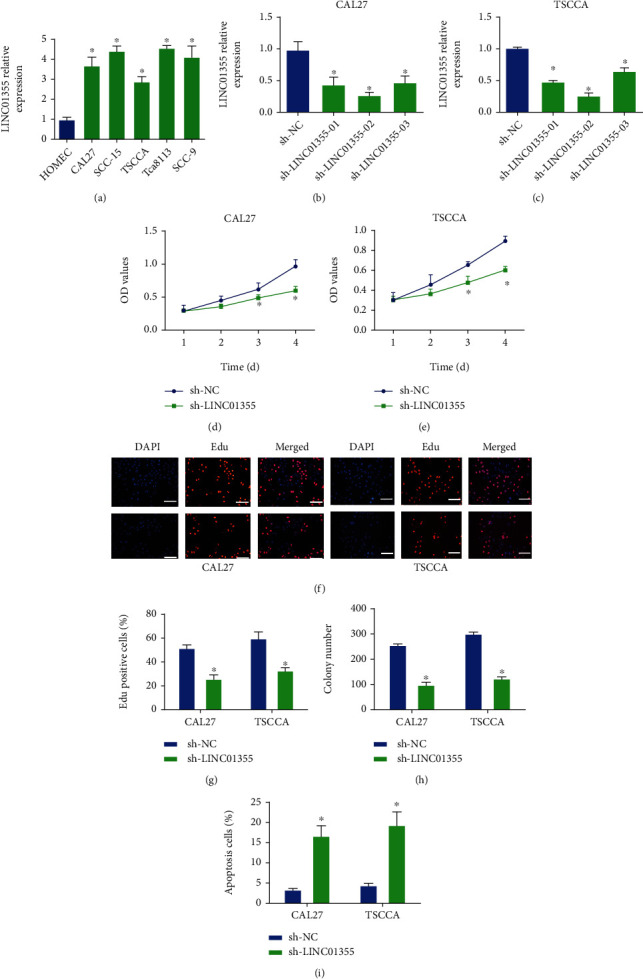
Loss of LINC01355 inhibited OSCC cell proliferation and induced cell apoptosis. (a) The expression of LINC01355 mRNA was detected by RT-qPCR analysis in HOMEC, CAL27, SCC-15, TSCCA, Tca8113, SCC-9 cells. (b, c) mRNA expression of LINC01355 was detected in CAL27 cells and TSCCA cells when transfected with several shRNAs of LINC01355. (d, e) The cell viability of CAL27 cells and TSCCA cells was analyzed after transfection with shRNA for LINC01355 for 1, 2, 3, and 4 days by CCK8 assay, respectively. (f, g) The cell proliferation of CAL27 cells and TSCCA cells was analyzed after transfection with the specific shRNA for LINC01355 by using EdU assay. (h) The cell colony formation capacity of CAL27 cells and TSCCA cells was analyzed using colony formation assay. (i) The cell apoptosis of CAL27 cells and TSCCA cells was analyzed using flow cytometry assay. ^∗^*p* < 0.05.

As exhibited in Figure 1(a), we found LINC01355 expression was upregulated in OSCC cells (CAL27, SCC-15, TSCCA, Tca8113, SCC-9) compared with HOMEC cells. These indicated increased LINC01355 might contribute to OSCC carcinogenesis. Then, CAL27 and TSCCA cell lines were used to carry out loss of function assays to explore the underlying mechanism of LINC01355 in OSCC. Specific shRNA of LINC01355 was transfected into CAL27 and TSCCA cells. In Figures 1(b) and 1(c), cells transfected with sh-LINC01355-02 abrogated mRNA expression level of LINC01355 as confirmed by qRT-PCR. CCK8 assays indicated the loss of LINC01355 obviously impaired the viability of OSCC cells (Figures 1(d) and 1(e)). In Figures 1(f) and 1(g), the EdU assay proved that OSCC cell proliferation was reduced by the loss of LINC01355. The results displayed that OSCC cells transfected with shRNA of LINC01355 demonstrated fewer colonies in Figure 1(h). In addition, we observed that cell apoptosis was expanded after silencing LINC01355 in comparison to the sh-NC cells (Figure 1).

### 3.2. Silence of LINC01355 Inhibited OSCC Cell Migration and Invasion

**Figure 2:**
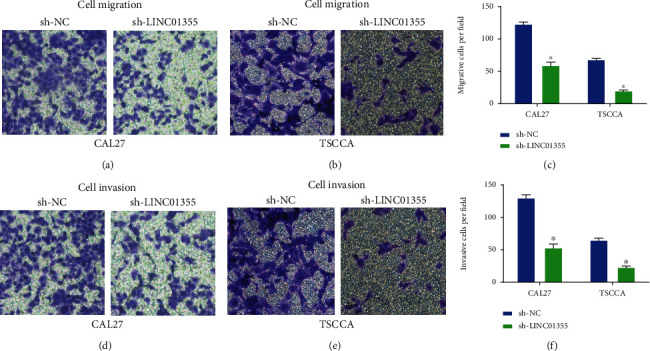
Loss of LINC01355 repressed OSCC cell migration and invasion. (a–c) Cell migration of CAL27 cells and TSCCA cells were analyzed after transfection with shRNA for LINC01355 using transwell migration assay. (d–f) Cell invasion of CAL27 cells and TSCCA cells were analyzed after transfection with the shRNA for LINC01355 using transwell invasion assay. ^∗^*p* < 0.05.

Next, we studied the function of LINC01355 in the motility of OSCC cells. As displayed in Figures 2(a)–2(c), the results implied that the LINC01355 knockdown significantly impaired the migration of CAL27 and TSCCA cells. As shown in Figures 2(d)–2(f), loss of LINC01355 led to reduce cell invasion.

### 3.3. Decreased LINC01355 Inhibited Tumor Growth and Triggered T Cell Infiltration in Xenograft

**Figure 3:**
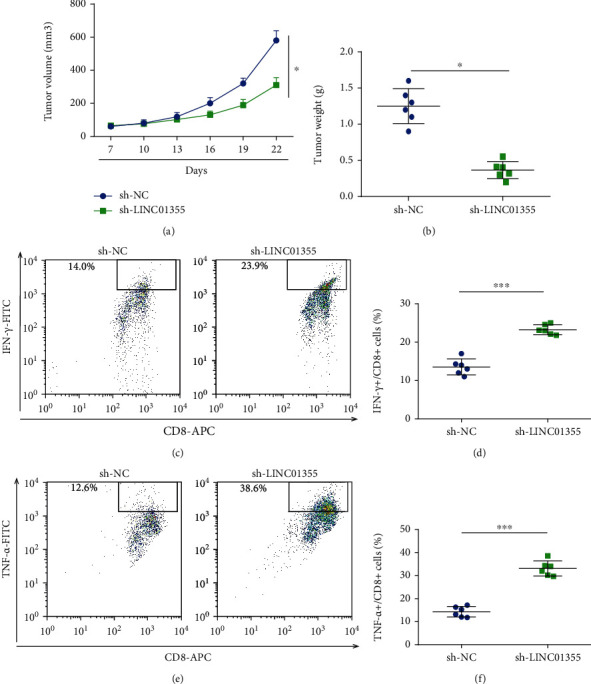
Knockdown of LINC01355 repressed tumor growth in vivo. BALB/c mice were challenged with 106 SCC-7 cells transfected with LINC01355 shRNA or NC subcutaneously. (a) Tumor volume. (b) Tumor weight was determined. (c, d) Frequency of tumor CD8+IFN-*γ*+ cells was analyzed using flow cytometry. (e, f) Frequency of tumor CD8+TNF-*α*+ cells was analyzed using flow cytometry. ^∗^*p* < 0.05; ^∗∗∗^*p* < 0.001.

Then, we determined the function of LINC01355 in vivo. LINC01355 knockdown SCC-7 cells were inoculated into BALB/c mice. Our data revealed the loss of LINC01355 exhibited antitumor effect in SCC-7 syngeneic models. We found that tumor volume was decreased time-dependently in Figure 3(a). Meanwhile, tumor weight was significantly reduced by the loss of LINC01355 as shown in Figure 3(b). To further prove the antitumor immunity regulated by LINC01355 shRNA, the percentage of tumor-infiltrating cytotoxic was evaluated. As indicated in Figures 3(c) and 3(d), the percentage of IFN-*γ*+CD8+ T cells was significantly induced by the knockdown of LINC01355. In addition, in Figures 3(e) and 3(f), we observed that the percentage of TNF-*α*+CD8+ T cells was enhanced by shRNA of LINC01355.

### 3.4. Downregulation of LINC01355 Inhibited the Cytotoxicity and Apoptosis and Increased the Proliferation of CD8+ T Cells

**Figure 4:**
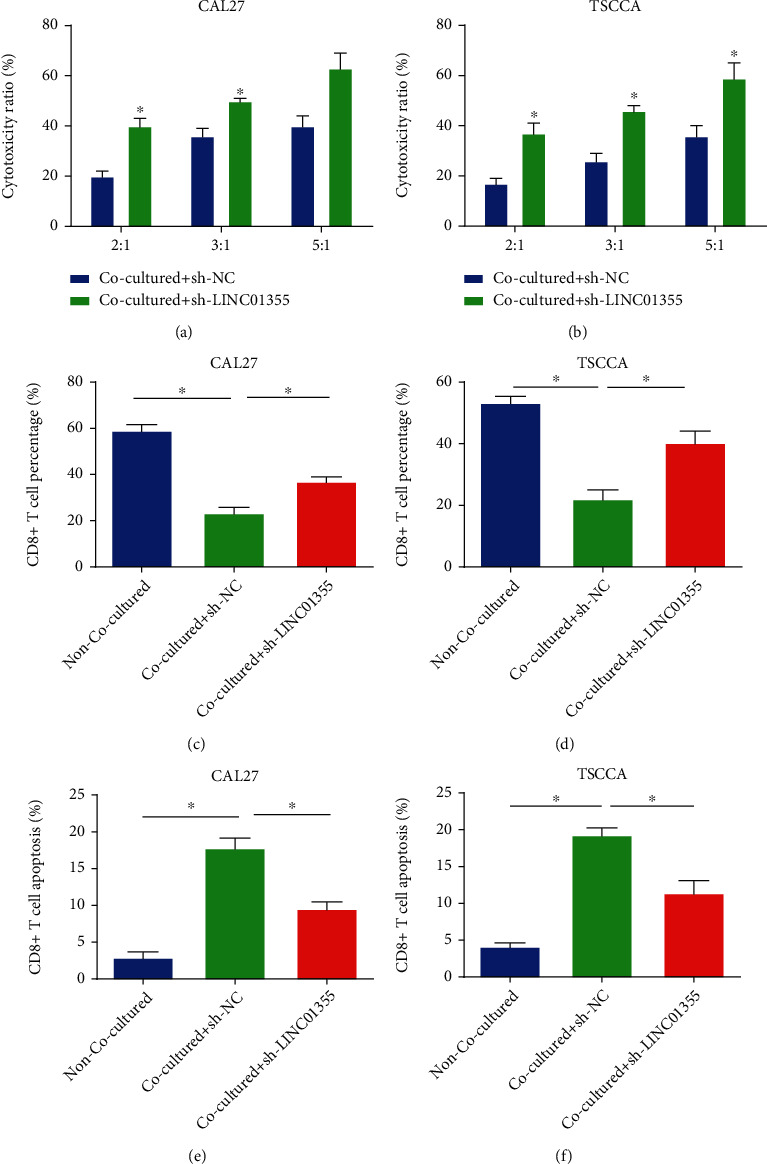
LINC01355 played a significant role in cytotoxicity, proliferation, and apoptosis of CD8+ T cells. The effector CD8+ T cells were obtained from peripheral blood of healthy donors and cocultured with target OSCC cells. (a, b) The cytotoxicity of CD8+ T cells was assessed using a LDH Kit. (c, d) The proliferation of CD8+ T cells was tested by CFSE assay. (e, f) The apoptosis of CD8+ T cells was tested by flow cytometry. ^∗^*p* < 0.05.

Functionally, we found that loss of LINC01355 in OSCC cells increased the cytotoxicity of CD8+ T cells in Figures 4(a) and 4(b). Loss of LINC01355 in OSCC cells induced the proliferation of CD8+ T cells as demonstrated in Figures 4(c) and 4(d). As shown in Figures 4(e) and 4(f), CD8+ T cell apoptosis was obviously repressed by loss of LINC01355.

### 3.5. Knockdown of LINC01355 Activated Notch Pathway in OSCC Cells

**Figure 5:**
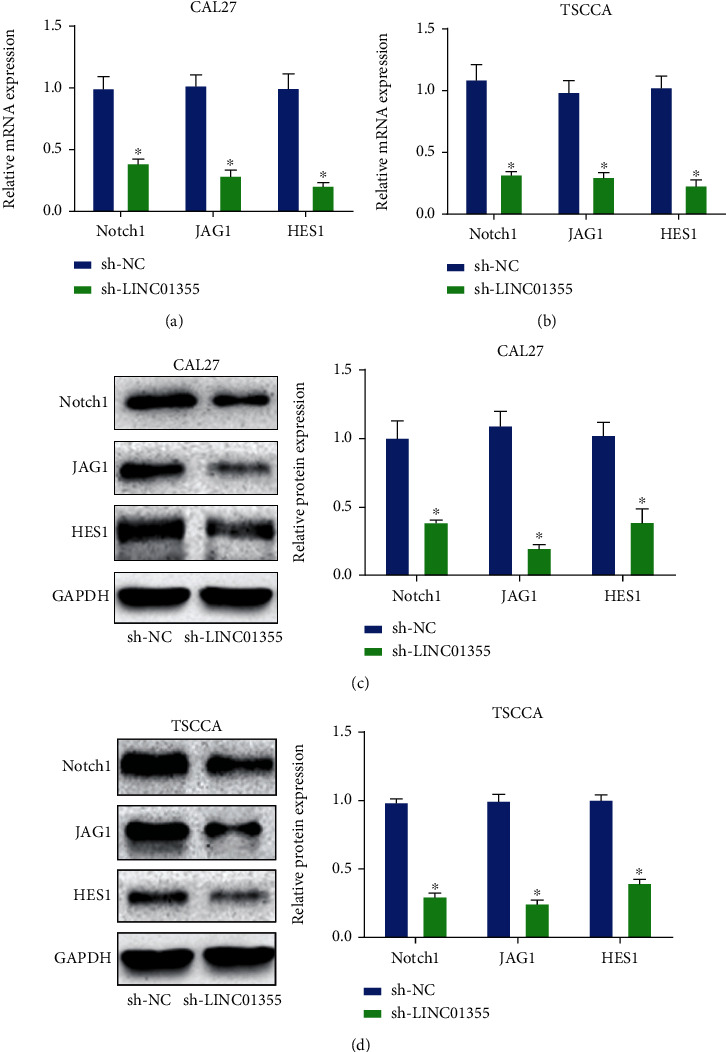
Knockdown of LINC01355 significantly repressed Notch signal in OSCC cells. (a, b) qRT-PCR analysis of Notch-1, JAG-1, HES-1 mRNA expression in OSCC cells transduced with LINC01355 shRNA or NC. (c, d) Western blotting analysis of Notch-1, JAG-1, HES-1 protein expression in OSCC cells transduced with LINC01355 shRNA or NC. ^∗^*p* < 0.05.

To explore the underlying mechanism by which LINC01355 modulates OSCC, we studied the Notch pathway. As shown in Figures 5(a) and 5(b), mRNA expression of Notch pathway-associated genes Notch-1, JAG-1, and HES-1 were downregulated in sh-LINC01355-transfected OSCC cells. In addition, protein expression of Notch-1, JAG-1, and HES-1 was also inhibited (Figures 5(c) and 5(d)).

### 3.6. Blocking Notch Signal Pathway Reversed the Effect of LINC01355 on OSCC

**Figure 6:**
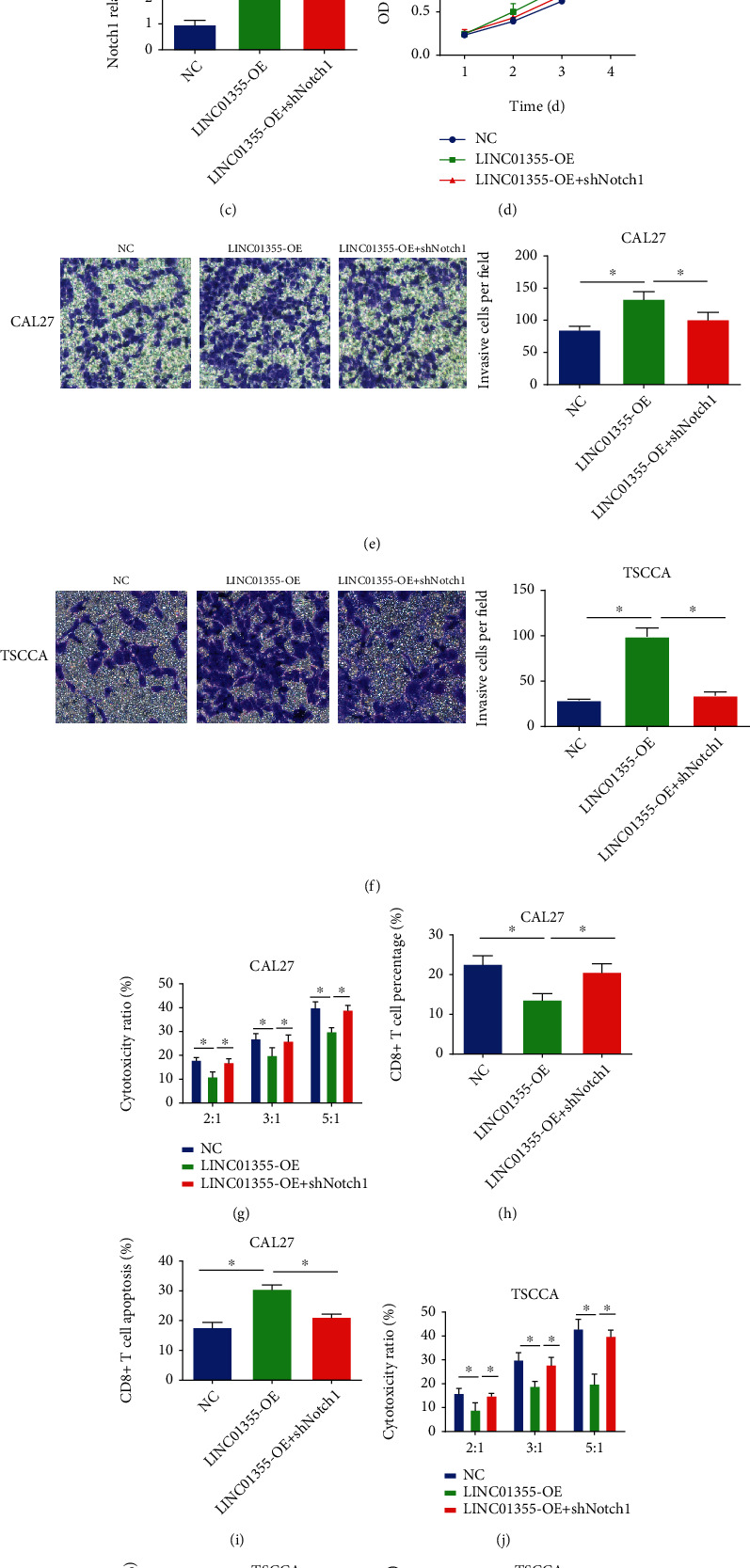
Knockdown of Notch1 inhibited the effect of LINC01355 on OSCC cells. (a) The Notch1 mRNA expression was assessed in CAL27 cells transfected with Notch1 shRNA or LINC01355 overexpression plasmid. (b) CAL27 cell viability was assessed by CCK-8 assay. (c) The Notch1 mRNA expression level was detected in TSCCA cells transfected with Notch1 shRNA or LINC01355 overexpression plasmid. (d) TSCCA cell viability was assessed using CCK-8 assay. (e, f) OSCC cell invasion. (g) The cytotoxicity of CD8+ T cells cocultured with CAL27 cells. (h) The proliferation of CD8+ T cells cocultured with CAL27 cells. (i) The apoptosis of CD8+ T cells cocultured with CAL27 cells. (j) The cytotoxicity of CD8+ T cells cocultured with TSCCA cells. (k) The proliferation of CD8+ T cells cocultured with TSCCA cells. (l) The apoptosis of CD8+ T cells cocultured with TSCCA cells. ^∗^*p* < 0.05.

Then, we assessed whether blocking the Notch pathway can reverse the function of LINC101355 on OSCC cell proliferation, invasion, and cytotoxicity of CD8+ T cells. As shown in Figures 6(a) and 6(b), knockdown of Notch-1 in CAL27 cells reversed cell viability induced by the overexpression of LINC01355. As indicated in Figures 6(c) and 6(d), the lack of Notch-1 in TSCCA cells reversed cell viability triggered by LINC01355. In Figures 6(e) and 6(f), Notch-1 shRNA reduced OSCC cell invasion capacity, which was increased by LINC01355. In Figures 6(g)–6(i), silencing Notch1 in CAL27 cells increased the cytotoxicity, proliferation, and depressed CD8+ T cell apoptosis. Inhibiting Notch1 in TSCCA cells manifested a similar phenomenon as shown in Figures 6(j)–6(l).

## 4. Discussion

LncRNAs are a class of more than 200 nt-long RNA molecules. Recently, a growing amount of lncRNAs are reported in pathological processes in many cancers [20, 21]. Meanwhile, the function of lncRNAs is involved in many processes, such as regulating X chromosome inactivation and chromatin remodeling [22–24]. LncRNAs are able to play essential roles in cancers. A recent research has indicated LINC01355 can inhibit breast cancer growth via transcriptional inhibition of CCND1 [25]. In this research, we observed that LINC01355 was increased in OSCC cells. Loss of LINC01355 reduced the growth of OSCC cells. We selected TSCCA and CAL-27 cell lines to investigate the function of LINC01355 in OSCC progression. In our future study, our data should be confirmed in more OSCC cell lines.

In general, many tumor cells contain antigens that can be identified by CD8+ T cells, to induce antitumor immune responses. Meanwhile, these CD8+ T cell responses are able to affect the survival of cancer cells. For instance, Lnc-Tim3 can exacerbate CD8+ T cell exhaustion through binding to Tim-3 and triggering nuclear translocation of Bat3 in liver cancer [26]. LncRNA GM16343 can induce IL-36*β* to modulate tumor microenvironment via CD8+ T cells [27]. LINC00301 can facilitate lung cancer progression and triggers an immune-suppressing microenvironment through regulating the HIF1*α* [28]. Immunomodulatory aspects have been reported in OSCC [29]. In our current study, loss of LINC01355 repressed the immune escape of tumor cells in OSCC. Decrease of LINC01355 inhibited tumor growth and triggered T cell infiltration in xenograft. CD8+ T cells demonstrate important roles in suppressing tumors. We observed that the percentage of IFN-*γ*+CD8+ T cells and TNF-*α*+CD8+ T cells were significantly induced by the knockdown of LINC01355. For another, loss of LINC01355 suppressed the cytotoxicity and apoptosis and enhanced the proliferation of CD8+ T cells.

The Notch signaling is an intercellular pathway, and it can regulate cell proliferation, apoptosis, and self-renewal [30–32]. The Notch pathway can exert an important role in tumor cells [32, 33]. Up to date, four Notch receptors (Notch1-4) with the corresponding ligand have been identified [34]. Notch signaling can induce the EMT process in OSCC cell lines under a hypoxic environment [35]. Activation of Notch is closely associated with OSCC progression [36]. Currently, we reported that lack of LINC01355 repressed Notch signaling and knockdown of Notch1 inhibited the effect of LINC01355 on OSCC cells.

Collectively, LINC01355 was increased in OSCC cells. In vitro studies confirmed the inhibition of LINC01355 suppressed the survival and migration/invasion of OSCC cells mediated by Notch. In addition, LINC01355 was found to activate the Notch pathway to help tumor cells evade T cell tumor immunity. In conclusion, we reported the role of LINC01355 in CD8+ T cell function and reported its roles in the immune escape of OSCC, which might offer an effective immunotherapy to treat OSCC.

## Figures and Tables

**Table 1:** Primers for real-time PCR.

Genes	Forward (5′-3′)	Reverse (5′-3′)
GAPDH	ACGGATTTGGTCGTATTGGGC	TTGACGGTGCCATGGAATTTG
LINC01355	CTGCTCTAGCCCCTAAAGATAG	GGATTCCAAATGACACATTCCT
Notch1	TGACTGTTCCCTCACTATGG	CACGTCTTGCTATTCCTCTG
JAG1	GGGAGAGTGATACTTGATGGG	CTCATTGTGGCTTTTGTGGAG
HES1	CTCCCGGCATTCCAAGCTAG	AGCGGGTCACCTCGTTC ATG

## Data Availability

The data used to support the findings of this study are included within the article.
